# Psychological Distress of Patients Experiencing Different Types of Road Traffic Injuries in Vietnam

**DOI:** 10.3390/ijerph17103429

**Published:** 2020-05-14

**Authors:** Chi Linh Hoang, Hai Minh Vu, Hai Quang Pham, Huong Lan Thi Nguyen, Linh Gia Vu, Bach Xuan Tran, Carl A. Latkin, Roger C. M. Ho, Cyrus S. H. Ho

**Affiliations:** 1 Center of Excellence in Behavioral Medicine, Nguyen Tat Thanh University, Ho Chi Minh City 700000, Vietnam; chi.coentt@gmail.com (C.L.H.); pcmrhcm@nus.edu.sg (R.C.M.H.); 2 Department of Trauma, Thai Binh University of Medicine and Pharmacy, Thai Binh 410000, Vietnam; vuminhhai777@gmail.com; 3 Institute for Global Health Innovations, Duy Tan University, Da Nang 550000, Vietnam; phamquanghai@duytan.edu.vn; 4 Faculty of Medicine, Duy Tan University, Da Nang 550000, Vietnam; 5 Faculty of Nursing, Duy Tan University, Da Nang 550000, Vietnam; 6 Center of Excellence in Evidence-Based Medicine, Nguyen Tat Thanh University, Ho Chi Minh City 700000, Vietnam; linh.coentt@gmail.com; 7 Institute for Preventive Medicine and Public Health, Hanoi Medical University, Hanoi 100000, Vietnam; bach.ipmph@gmail.com; 8 Bloomberg School of Public Health, Johns Hopkins University, Baltimore, MD 21205, USA; carl.latkin@jhu.edu; 9 Department of Psychological Medicine, Yong Loo Lin School of Medicine, National University of Singapore, Singapore 119228, Singapore; 10 Institute for Health Innovation and Technology (iHealthtech), National University of Singapore, Singapore 119077, Singapore; 11 Department of Psychological Medicine, National University Hospital, Singapore 119074, Singapore; cyrushosh@gmail.com

**Keywords:** psychological distress, non-fatal injuries, road traffic injuries

## Abstract

Road-related injuries are often catastrophic, and the eighth leading cause of all-aged mortality. While psychological problems, including anxiety, driving phobia, and post-traumatic stress have been found to be common among injured survivors, the literature in this area is still limited. This study aimed to evaluate the prevalence of distress between different types of road injuries among 413 patients in Thai Binh hospitals from October to December 2018. The Kessler Psychological Distress Scale (K6) was used to assess mental health status. Sociodemographic and clinical characteristics were also collected. The results of Multiple Logistic and Tobit regression models were utilized. Psychological issues were found in 13.8% of the participants. In terms of K6 profile, nervous, restless/fidgety, and “everything was an effort” were the three most frequently endorsed aspects. Having soft-tissue injuries had a 0.32-time lower likelihood of psychological distress compared to those having other injuries. Additionally, patients who were diagnosed with fractures were 4.5-times more likely to report psychological distress. Our finding highlights the need for psychological screening to reduce disabilities associated with non-fatal injury related to road traffic crashes.

## 1. Introduction

With an increased number of vehicles, road-related injuries have been identified as the eighth leading cause of all-aged mortality and might be up to the third top cause of disability in 2020 [1]. Annually, more than 1.35 million people die, and about 50 million people suffer from long-lasting injuries as a result of road traffic crashes [1]. Approximately 80% of road traffic death occurs in middle-income countries, which is due in part to the population explosion and increase in the number of motor vehicles [1]. A recent review showed that injured survivors might undergo a burden of disease, including direct cost (i.e., the cost of illness) and indirect cost (loss of productivity and the care provided to disabled family members) [2].

At the time of road traffic crashes, the interplay of gravitational force and velocity of the vehicle lead to fatal injuries such as traumatic brain injuries, spinal cord, fracture, and other multiple injuries (i.e., significant impact to organs and damage resulting in hypovolemic, cardiogenic shock) [3]. In addition, the consequence of non-fatal injuries is also considered not only in terms of physical injury but also psychological issues, which are often neglected in research as mortality rate is a common indicator to evaluate global health progress. Previous studies found that the cost of illness, including medical and rehabilitation costs, might double if the elevated rates of psychological distress are considered in injured people [4]. Another study pointed out that psychological problems such as anxiety, driving phobia, and post-traumatic stress are identified in more than half of people involved in road traffic crashes [5,6,7]. If post-traumatic stress disorder is not detected and left untreated, it impacts physical recovery, resilience, and other activities. This is reflected in chronic conditions and other behavior disorders, including violent behavior and substance abuse [8,9,10,11,12]. Therefore, early mental health screening and interventions have been recommended to reduce the burden of road traffic crashes [5,13].

In Vietnam, during a period of 10 years—from 2003 to 2013—road traffic was the cause of nearly 45% of all injuries-related deaths, and was expected to rise as the second leading cause of disability in 2020 [2]. While new policies on strengthening road safety management contribute to the decreasing trend of road accident-related deaths to below 10,000 per year, the prevalence of road injuries in Vietnam remains high. This can be explained by the fact that preventing non-fatal injuries is not the road safety target in many low- and middle-income countries [1]. Additionally, many high-income countries focus on the health burden of non-fatal injuries, the long-term disability of road traffic injuries; however, it does not attract adequate attention in developing countries even though more than 80% of such events occur in these countries [2]. Therefore, our study aims to evaluate the prevalence of psychological distress and the associated risks of psychological distress with different types of non-fatal traffic injuries in Vietnam.

## 2. Materials and Methods

### 2.1. Study Setting and Location

This study is part of a project that aimed to assess the health status of patients in six hospitals in Thai Binh province from October to December 2018. Convenience sampling was used to recruit 413 from 430 eligible patients based on the following criteria: (1) being 18 years old or above; (2) hospitalized due to road traffic crashes; (3) willing and able to have a conservation with the data collectors. Those who were unable or unwilling to answer the questionnaire due to severe injuries or those with a history of psychological issues were excluded from the study.

Trained collectors (i.e., medical doctors and nurses in departments) approached and introduced the participants to a face-to-face interview. After giving their informed consent, the participants were invited to a private counseling room to ensure privacy. The data was collected by using a self-administered paper-based questionnaire. In this study, if the participants were unable to fill the questionnaire by themselves, the trained collectors read and supported them to complete the questionnaire. Any questions that the participants could not understand clearly were clarified by the interviewers.

### 2.2. Measurements and Instruments

A structured questionnaire was formed to collect data related to demographic information, characteristics of injuries, and psychological distress patterns. In order to evaluate the validity and reliability of the designed tool, about 10 volunteers participated in a pre-pilot before revising and conducting in the survey.

#### 2.2.1. Socioeconomic Characteristics

We collected general information, including socioeconomic variables (gender, age, marital status, level of education, career, monthly income) and health insurance status.

#### 2.2.2. Injury-Related Characteristics

Information related to the injuries was exported from medical reports and divided into eight categories: soft-tissue injury, soft-tissue wound, hand injury, traumatic brain injury, maxillofacial wound, spinal wound, chest wound, and fracture. In particular, soft tissue injuries refer to minor abrasions and bruises to major trauma such as tendon rupture, sprain, and muscle strains [14]. Soft tissue wound is defined as any disruption of skin integrity, mucous membrane, or organ tissue. As hand surgeons require advanced techniques (i.e., microsurgical reattachment or microsurgical reconstruction of soft tissues and bone, nerve reconstruction, and surgery) to improve the function of upper limbs, hand injury was classified as a specialized trauma. Other types of injury were classified according to the site of injury.

Additionally, the collector also recorded the characteristics related to injury treatment consisting of a number of injuries/wounds, type of surgery, number of treatments, and length of hospitalization.

#### 2.2.3. Psychological Distress

To measure psychological distress, we used the Kessler scale (K6), which includes six items to screen for mental health conditions [15]. Each question has a severity score from 0 to 4, and the total score ranged from 0 to 24. A value > 5 was considered as the cut-off point to determine psychological distress in this study. The internal consistency reliability of this instrument was acceptable with Cronbach’s alpha = 0.74.

#### 2.3. Statistical Analysis

Frequencies, percentages, means, and standard deviations (SD) were presented for descriptive statistics. A stacked bar chart was used to present the domains score of Kessler. To identify associated factors relating to Kessler score and mental status, socioeconomic factors (i.e., age, gender, level of education, occupation, income, marital status, living areas) and the information regarding treatment (i.e., length of hospitalization, drug administration, correction/pundle, soft-tissue surgery, osteosynthesis, and other treatments) was included in the multivariable logistic regression model STATA 15.0 (Stata Corp. LP, College Station, TX, USA). We obtained the estimates and 95% CIs from the unadjusted and adjusted models, along with the *p*-values.

### 2.4. Ethical Approval

The protocol was evaluated and approved by the Institutional Review Board of Thai Binh University of Medicine and Pharmacy. All the patients were carefully explained the purpose and relevant risks of the survey before obtaining a written informed consent. Participants’ information was used for research purposes only (ethics approval code: 7642/HĐĐĐ).

## 3. Results

Table 1 provides the general information of the participants. Overall, most of the participants were 18–40 years old (42.1%); males made up more than 60% of the sample, and the majority lived with their spouse/partner (72.4%). More than half of the participants were blue-collar workers (55%) and lived in urban areas (85.5%). Approximately 15% of the respondents in both genders had high levels of psychological distress symptoms. There was a significant difference in psychological prevalence between people living alone and those living with a spouse/partner (20.2% vs. 11.4%, *p* < 0.05). In terms of occupation, white-collar workers had the lowest rate of psychological issue compared to those who held other jobs (8%).

The classification of injury and other medical characteristics are indicated in Table 2. Soft tissue injuries and fractures were mentioned most frequently, with 117 and 144 patients, respectively. About 16% of the patients having muscle-skeleton injuries, including traumatic brain injury, maxillofacial wounds, and soft-tissue injuries reported distress symptoms. Soft-tissue surgery also had the highest percentage of reporting distress—20.8%—compared to other interventions.

**Figure 1:**
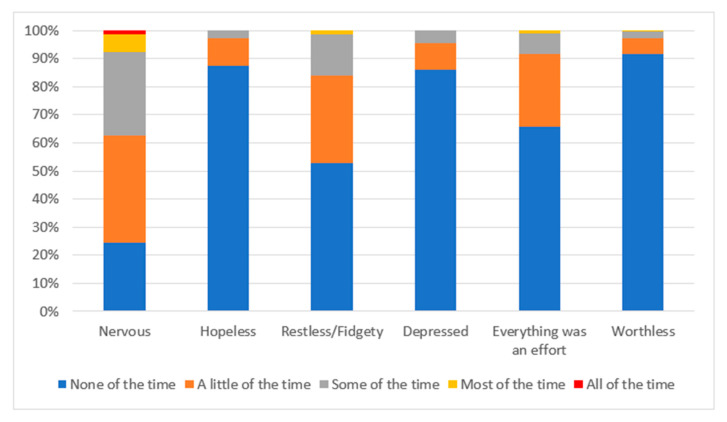
Profile of six-item Kessler Psychological Distress Scale (K6) responses.

The average K6-score was 2.7 (SD = 2.6). Figure 1 presents the K6 profiles in detail. In particular, nervous, restless/fidgety, and “everything was an effort” were the three most frequently reported aspects of psychological distress among the participants.

The multivariate regression model is presented in Table 3. After adjustment for the confounders (i.e., socioeconomic factors and comorbidities), patients with soft-tissue injuries had a 0.32-time lower likelihood of psychological distress compared to those having other injuries (OR = 0.32, 95%CI = 0.11; 0.92). Additionally, patients who were diagnosed with fractures were 4.5-times more likely to report distress (OR = 4.5, 95%CI = 1; 20.12).

## 4. Discussion

This study offers an insight into psychological distress in patients with road traffic injuries. A high prevalence of psychological distress among people suffering from road traffic injuries were reported in our result. Patients who suffered fractures or required orthopedic surgery were associated with a higher risk of psychological distress. Thus, our finding highlights the necessity of psychological support from health professionals to improve psychosocial and quality of life outcomes.

Previous literature has established a link between injuries and psychological distress [8,13,16,17]. It can be seen that unintentional injuries (i.e., automobile crashes, industrial accidents, home accidents) not only increased the risk of psychological issues, but were also associated with illicit drug use, alcohol use, and violent behavior [9,10,18,19]. In this study, the number of patients having psychological distress was found to be higher than the previous studies for unintentional injury survivors in the United States, in which 3.7% and 5.3% of those reported moderate and severe levels of psychological distress, respectively [17]. Additionally, in a cohort study conducted with 87,151 distance learning students in Thailand, more than one-third of traffic-injury members were found to qualify for psychological treatment [16]. As all participants in this study were inpatients, receiving direct support from health professionals could be a reason for the lower level of distress. The results lend further credence to earlier research, citing that nervous and restless/fidgety were two major psychological characteristics among the researched population. Indeed, blue-collar workers accounted for more than half of the participants, and worrying about the loss of their life, loss of breadwinner role, and becoming a burden for their family were mentioned as the main concerns among these patients [20].

Our results indicated that patients having only soft-tissue injuries were likely to have lower psychological distress scores than other road-related injury patients. Once a crash occurs, the passengers/drivers have to deal with not only life-threatening problems, but also the trauma of the event. Therefore, when patients suffer from soft-tissue injuries, they might release their fear of complications and reduce the risk of post-accident psychological distress. Other scholars, however, have documented that the higher level of the severity of trauma at the baseline is not always associated with psychological symptoms [21]. This is reflected in the increased level of distress among patients having soft tissue injuries, which is presented as the most common of minor transport injuries [22], and many chronic consequences arise from relatively minor injuries [23] and thus may lead to more complex problems requiring multi-faceted management, such as mental issues, in comparison with other injuries [22,24]. In this setting, we could not clarify the influence of different levels of injuries and psychological symptoms because of the limited scope of the study compared to previous researches [24]. These results suggest the importance of interventions to promote quality of care for patients with soft-tissue injuries after a traffic crash. Further road safety programs should also be focused on reducing the burden of non-fatal injury, rather than attempting to reduce only the mortality of road traffic crashes.

We also confirmed that having a fracture was significantly associated with a higher risk of distress. This result is consistent with previous studies, in which fracture was found to be the most common predictor of pain among elective surgery patients and results in a higher risk of psychological distress [23,24]. During treatment, most of the fracture cases are associated with multi-injuries; hence, even though the surgery is successfully performed, it still requires complex treatment and long-term rehabilitation. In fact, due to the majority of accidents occurring among young adults or people of working age, the most common concern is often the loss of productivity and fear of pain both before and after surgery [21,25]. Our findings also reported that patients being single or having longer hospitalization had a significantly higher prevalence of distress, compared to the others. Among all types of non-fatal road injuries, traumatic brain injury places the heaviest burden on patients [26] with multidimensional complications and long-term hospitalization [27,28,29,30]. In this study, we did not find an association between traumatic brain injury and the prevalence of psychological distress, which were found in a previous study, as severe cases were excluded from our study. Further study, therefore, should evaluate the prevalence of psychiatric disorders (i.e., anxiety and depression) stemming from traumatic brain injuries [29].

To improve the outcome of critical care for injured patients, our study suggests that local health professionals integrate early interventions and consultation not only for patients with physical problems, but also those with mental health issues. There are several limitations in this study. Firstly, we did not collect information related to the type of road user; for instance, pedestrians, drivers, etc., which might be necessary to suggest further road safety interventions. Secondly, there was a lack of clinical variables consisting of the type of surgery and severity of the injury. However, the majority of participants had mild injuries, while others with critical conditions were transferred to another hospital. Future studies should be designed to identify associated factors with the risk of distress among injured survivors. Our study used a convenience method to recruit the participants, so selection bias is also one of our limitations. Lastly, we conducted a cross-sectional design, which does not allow us to identify the causal relationship between psychological distress and predicting factors.

## 5. Conclusions

Our study shows a high prevalence of distress in people after a traffic crash. The type of injury, including soft-tissue injuries and fractures, has a correlation with mental health issues. Integrating screening and support from local health professionals need to be implemented to provide comprehensive care and reduce the long-term disability for injured survivors.

## Figures and Tables

**Table 1:** Socioeconomic status of the respondents.

Characteristic	Not Having Psychological Distress		Having Psychological Distress		Total		*p* Value
	n	%	n	%	n	%	
**Total**	356	86.2	57	13.8	413	100	
**Gender**							
Female	136	85.0	24	15.0	160	38.7	0.57
Male	220	87.0	33	13.0	253	61.3	
**Education**							
Less than high school	161	83.0	33	17.0	194	47.0	0.08
High school and above	195	89.0	24	11.0	219	53.0	
**Marital status**							
Single	91	79.8	23	20.2	114	27.6	0.02
Live with spouse/partner	265	88.6	34	11.4	299	72.4	
**Occupation**							
Freelance	74	87.1	11	12.9	85	20.6	0.69
White-collar worker	23	92.0	2	8.0	25	6.1	
Blue-collar worker	196	86.3	31	13.7	227	55.0	
Others	63	82.9	13	17.1	76	18.4	
**Location**							
Rural	53	88.3	7	11.7	60	14.5	0.60
Urban	303	85.8	50	14.2	353	85.5	
**Household monthly income**							
Poorest	99	83.9	19	16.1	118	28.6	0.53
Poor	60	85.7	10	14.3	70	16.9	
Normal	52	86.7	8	13.3	60	14.5	
Rich	85	91.4	8	8.6	93	22.5	
Richest	60	83.3	12	16.7	72	17.4	
	**Median**	**IQR**	**Median**	**IQR**	**Median**	**IQR**	
**Age** (years)	45	31–60	47	27–58	45	30–59	0.56

**Table 2:** Psychological distress according to types of injury and treatment.

Characteristic	Not Reporting Psychological Distress		Reporting Psychological Distress		Total		*p* Value
	n	%	N	%	n	%	
**Health problems**							
Soft tissue injuries	99	84.6	18	15.4	117	100	0.56
Hand injury	16	88.9	2	11.1	18	100	0.74
Traumatic brain injury	64	83.1	13	16.9	77	100	0.39
Maxillofacial wound	21	84	4	16	25	100	0.74
Spinal wound	16	88.9	2	11.1	18	100	0.74
Chest wound	12	100	0	0	12	100	0.16
Fracture	123	85.4	21	14.6	144	100	0.16
Soft tissue wound	80	88.9	10	11.1	90	100	0.40
**Number of wounds/injuries**							
1	261	85.6	44	14.4	305	100	0.71
≥2	81	87.1	12	12.9	93	100	
Treatment							
Drug administration	180	87	27	13	207	100	0.65
Correction/pundle	49	87.5	7	12.5	56	100	0.76
Soft-tissue surgery	61	79.2	16	20.8	77	100	0.05
Osteosynthesis	61	85.9	10	14.1	71	100	0.94
Tendon joint surgery	8	100	0	0	8	100	0.25
Maxillofacial surgery	5	100	0	0	5	100	0.37
Others	41	82	9	18	50	100	0.36
**Number of types of treatment**							
1	307	87	46	13	353	100	0.20
≥2	46	80.7	11	19.3	57	100	
**Comorbidity diseases**							
0	207	87.3	30	12.7	237	100	0.20
1	109	83.8	21	16.2	130	100	
≥ 2	40	87	6	13	46	100	
	**Median**	**IQR**	**Median**	**IQR**	**Median**	**IQR**	
**Length of hospitalization** (days)	7	4–10	9	5–12	7	4–10	0.05

**Table 3:** Factors associated with Kessler score and having psychological distress.

Health Problems	N	Unadjusted OR	95% CI	Adjusted OR	95% CI
**Soft tissue injuries** (Yes vs. No)	257	1.2	(0.65; 2.19)	0.32 **	(0.11; 0.92)
**Soft tissue wound** (Yes vs. No)	257	0.29	(0.06; 1.46)	0.77	(0.17; 3.45)
**Hand injury** (Yes vs. No)	257	0.77	(0.17; 3.45)	0.77	(0.07; 7.91)
**Traumatic brain injury** (Yes vs. No)	257	1.35	(0.69; 2.65)	2.16	(0.67; 7.02)
**Maxillofacial wound** (Yes vs. No)	257	1.2	(0.4; 3.65)	0.89	(0.22; 3.68)
**Spinal wound** (Yes vs. No)	257	0.77	(0.17; 3.45)	0.89	(0.1; 8.31)
**Fracture** (Yes vs. No)	257	1.11	(0.62; 1.98)	4.50 **	(1; 20.13)
**Multi-wound/injury** (vs. 1)	173				
2	69	0.8	(0.38; 1.67)	1.69	(0.34; 8.42)
≥2	9	0.45	(0.16; 1.28)	1.04	(0.15; 7.2)

** *p* < 0.05.
